# The landscape of pediatric genetic white matter disorders at a tertiary referral hospital in Upper Egypt and the report of 31 novel variants

**DOI:** 10.1186/s13052-025-02031-6

**Published:** 2025-06-13

**Authors:** Mahmoud M. Noureldeen, Maha S. Zaki, Karima Rafat, Mohamed S. Abdel-Hamid, Aida M. S. Salem

**Affiliations:** 1 Department of Pediatrics, Faculty of Medicine, Beni-Suef University, Beni-Suef, Egypt; 2 Clinical Genetics Department, Human Genetics and Genome Research Institute, National Research Centre, Cairo, 12311 Egypt; 3 Medical Molecular Genetics Department, Human Genetics and Genome Research Institute, National Research Centre, Cairo, Egypt

**Keywords:** Leukodystrophies, Genetic leukoencephalopathies, Genetic white matter, Beni-Suef, Egypt

## Abstract

**Background:**

Leukodystrophies (LDs) and genetic leukoencephalopathies (GLEs) encompass the spectrum of genetic white matter disorders (GWMDs). Despite their clinical significance, limited studies have investigated GWMDs in Egypt. Therefore, this study aimed to characterize pediatric patients diagnosed with GWMDs in the Beni-Suef Governorate, Upper Egypt.

**Methods:**

We reviewed the records of patients diagnosed with GWMDs who presented over five years to the pediatric neurology clinic of a tertiary care hospital in Beni-Suef Governorate, Upper Egypt. The study included 142 patients aged < 18 years diagnosed with GWMD confirmed by brain imaging, metabolic, and/or molecular genetic testing. Patients were classified as LDs or GLEs per the 2015 Global Leukodystrophy Initiative Consortium (GLIA) criteria.

**Results:**

Fifty-six cases were identified to have LDs, while 86 were classified as GLEs. Metachromatic leukodystrophy (MLD) was the most common LD (13 patients), followed by megalencephalic leukoencephalopathy with subcortical cysts (MLC) (10 patients). The most common GLEs were lysosomal storage disorders (LSDs) (22 patients,) followed by Cockayne syndrome (11 patients), along with other miscellaneous disorders. The cumulative incidence of GWMDs in children under 18 was estimated at 10.8 cases per 100,000 population during the five-year study period. Thirty-one novel variants were identified, comprising 10 for LDs and 21 for GLEs. The mortality rate was 39.3% and 22.1% among patients with LDs and GLEs, respectively.

**Conclusions:**

This study presents the first cohort of GWMDs reported from the Beni-Suef Governorate, Upper Egypt. The study provides significant data regarding regional etiological patterns, clinical trajectories, and molecular profiles. Additionally, the study findings provide a foundational framework for establishing a national GWMD registry and inform future diagnostic and therapeutic strategies.

## Background

Genetic white matter disorders (GWMDs) are heritable conditions characterized by structural or functional abnormalities in the central nervous system’s (CNS) white matter (WM), often resulting in progressive neurological impairment [1]. Vandever et al. proposed a classification system to standardize research across heterogeneous populations. They categorized GWMDs into two distinct groups; the first group comprises LDs, characterized by primary pathology of myelin or glial cells (e.g., oligodendrocytes and astrocytes). The second group includes GLEs involving hereditary white matter abnormalities with systemic or grey matter predominance [2]. In 2017, Van der Knaap and Bugiani proposed an alternative classification system grounded in cellular pathology, including categories such as astrocytopathy and leuko-axonopathies [3]. This approach improves the mechanistic understanding of GWMDs; however, its dependence on histopathologic specificity complicates its application in population-based studies, where clinical and radiologic phenotypes are prioritized for standardization.

The childhood incidence rates of GWMDs vary dramatically across populations, underscoring the influence of genetic diversity, consanguinity, and diagnostic practices. Studies report incidence rates ranging from 1.2/100,000 live births in Washington DC (2004–2009) [4] to 30/100,000 in Finland [5]. Other studies have reported the incidence of LDs to be approximately 3.1/100.000 in the UK [6], 13/100000 in the United States [7], and 2.2 to 3.1/100000 in Saudi Arabia [8]. In Egypt, where consanguinity rates exceed 30% [9], GWMDs are likely underdiagnosed due to fragmented data and the absence of a national registry. A recent study by Fateen et al. estimated the incidence of metachromatic leukodystrophy (MLD) at 1.6/100,000 [10], yet broader GWMD epidemiology remains uncharacterized.

Diagnosis integrates clinical evaluation with magnetic resonance imaging (MRI) pattern recognition, where radiologic hallmarks—such as periventricular hyperintensities, frontal predominance, or brainstem involvement—guide targeted genetic testing [11]. In cases lacking distinct MRI patterns, whole-exome sequencing (WES) serves as a critical diagnostic tool [12]. Because whole-exome sequencing can bypass the diagnostic dilemma many patients experience, it may emerge as the preferred approach for GWMD diagnosis [13]. However, its cost and potential for false-negative results remain limitations [14]. Proceeding to whole-genome sequencing (WGS) in unresolved cases significantly improves diagnostic outcomes for GWMDs [15]. Early diagnosis and intervention are crucial, particularly in disorders where disease progression can be halted through stem cell transplantation (SCT), such as metachromatic leukodystrophy (MLD), X-linked adrenoleukodystrophy (ALD), and Krabbe disease (KD) [16].

The elevated consanguinity rates in Egypt [9] present a significant opportunity for advancing GWMD research. However, prior Egyptian studies either lacked adoption of the standardized 2015 GLIA classification [17, 18] or focused on selected disorders (e.g., MLD) [19]. Moreover, no genetic or epidemiological data are available for the Beni-Suef Governorate. The current study addresses these gaps by applying the GLIA framework to estimate GWMD incidence, delineate clinical-radiologic-genetic profiles, and identify consanguinity-driven novel variants. This study integrates epidemiologic and molecular insights, establishing a foundation for national registries, improving genetic counseling, and guiding therapeutic access (e.g., SCT) in high-risk populations, thereby advancing global GWMD research.

## Methods

### Study design

This retrospective cohort study was conducted at Beni-Suef University Hospital’s pediatric neurology outpatient clinic, the exclusive tertiary care center for pediatric neurology in Beni-Suef Governorate, Upper Egypt. The Research Ethics Committee of Beni-Suef University’s Faculty of Medicine approved the study protocol (Approval No. FMBSUREC/07072024/Nour Eldeen) and the Medical Research Ethics Committee of the National Research Centre, Egypt. Data were retrieved from medical records of patients diagnosed with GWMDs between January 2019 and December 2023. Written informed consent was obtained from the parents or legal guardians of all pediatric participants before enrollment. The study adhered to the ethical principles of the Declaration of Helsinki.The reporting of this observational cohort study adheres to the Strengthening the Reporting of Observational Studies in Epidemiology (STROBE) guidelines.

### Participants

**Fig. 1:**
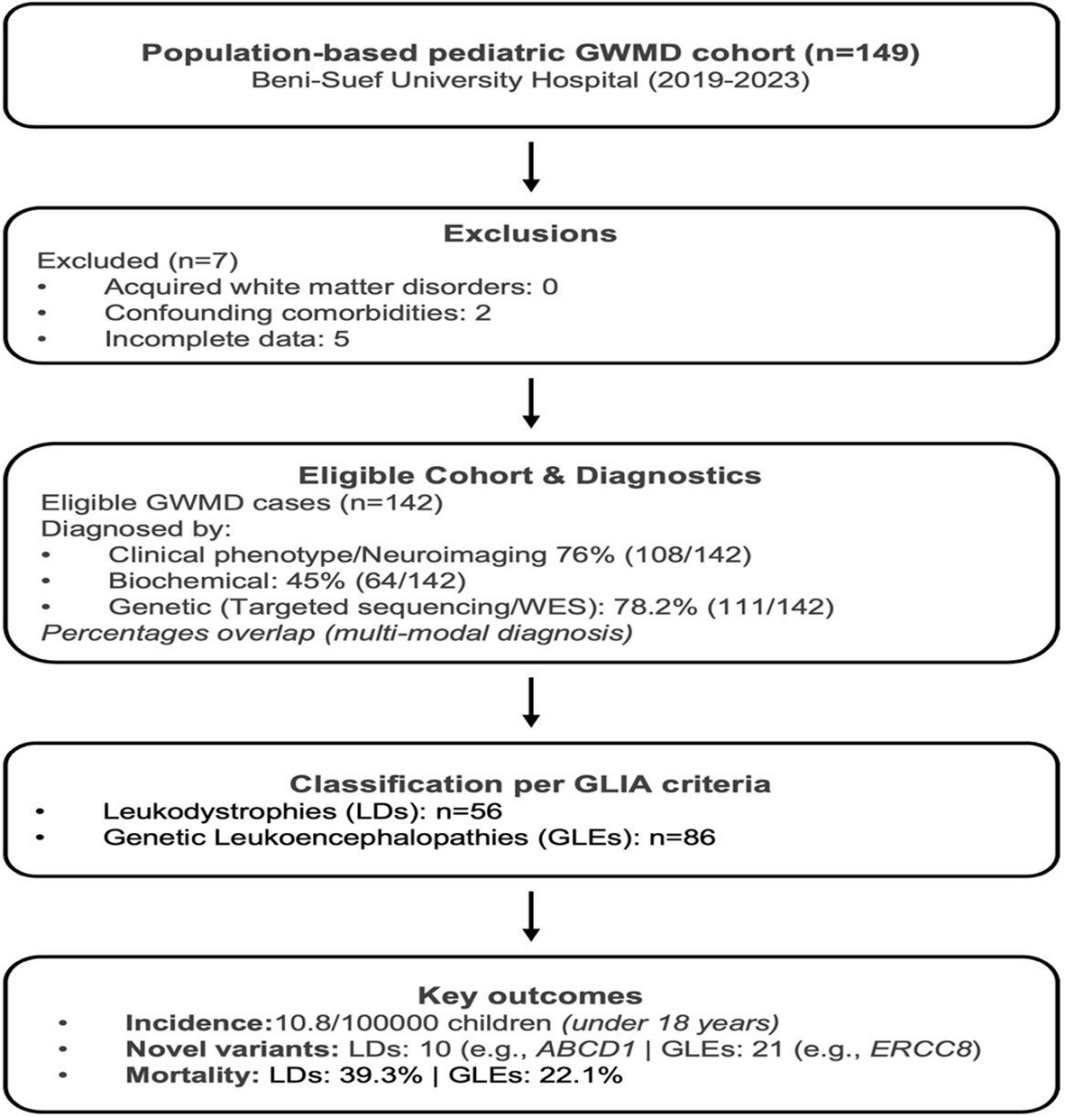
Flow diagram of the pediatric genetic white matter disorder cohort study at Beni-Suef University Hospital, Egypt (2019–2023). Abbreviations: GWMD, genetic white matter disorder; WES, whole-exome sequencing

Eligibility criteria encompassed pediatric patients under 18 years of age with a confirmed GWMD diagnosis, supported by at least one of the following: (1) a distinctive clinical phenotype (e.g., progressive motor or cognitive decline), (2) neuroimaging evidence (MRI/CT findings consistent with leukodystrophy or hypomyelination), and/or (3) biochemical or molecular genetic confirmation (e.g., pathogenic variants in GWMD-associated genes or abnormal enzymatic activity). Exclusion criteria comprised acquired white matter disorders (e.g., multiple sclerosis, post-infectious demyelination, toxic leukoencephalopathies), significant comorbidities confounding neurological or imaging findings (active malignancy, prior CNS-directed radiation/chemotherapy, primary cardiovascular or rheumatic diseases), and cases with incomplete clinical or imaging data. As a population-based retrospective cohort, all eligible GWMD cases diagnosed during the 5-year study period were included (*n* = 142). This population-based study systematically identified all GWMD cases in the region during the study period, prioritizing complete case ascertainment over traditional sample size calculations.This methodology aligns with other epidemiological studies for GWMDs [5]. Of 149 initially identified cases, 7 were excluded (2 due to confounding comorbidities, 5 with incomplete data), yielding 142 cases analyzed through clinical, biochemical, and molecular diagnostics (Fig. 1). This comprehensive approach enabled full characterization of the condition’s clinical spectrum and outcomes in our population.

### Evaluations and data collection

The pediatric neurology and clinical genetics team performed all clinical assessments using standardized proformas, requiring 60–90 min per patient*.* Data collection included demographic profiles, developmental histories, disease progression timelines, seizure semiology, and neurological examination findings. Final diagnoses required agreement between two experienced clinicians. All neuroimaging studies were performed using standardized protocols at Beni-Suef University Hospital. MRI examinations utilized a 1.5 Tesla Siemens scanner, incorporating specific sequences such as T1-weighted, T2-weighted, fluid-attenuated inversion recovery (FLAIR), and diffusion-weighted imaging (DWI). A non-contrast CT was performed on a 64-slice Siemens scanner for suspected calcifying leukoencephalopathies. MR spectroscopy (MRS) was utilized in specific instances to evaluate metabolic profiles, such as increased N-acetylaspartate (NAA) levels in Canavan disease. All images underwent clinical interpretation by the hospital’s neuroradiology service, and official reports were systematically integrated into the study datasets.

All biochemical analyses were performed following standardized quality control protocols. Tandem mass spectrometry (TMS) serves as a diagnostic tool for aminoacidopathies, including phenylketonuria (PKU) and maple syrup urine disease (MSUD). Urine organic acid analysis using gas chromatography-mass spectrometry (GC–MS) identified organic acidemias, including glutaric aciduria type I (GA1) and methylmalonic acidemia (MMA). Additionally, elevated urinary NAA detected by GC–MS confirmed the presence of Canavan disease. Enzyme activity assays are essential for the diagnosis of certain LDs: arylsulfatase A (ASA) activity indicates MLD, while galactocerebrosidase (GALC) activity is indicative of KD. Liquid chromatography-tandem mass spectrometry (LC–MS/MS) was used for plasma VLCFA profiling (X-ALD), homocysteine quantification (hereditary homocystinuria), and urinary sulphocysteine/xanthine analysis (molybdenum cofactor deficiency, MoCD; sulfite oxidase deficiency, SOD).

Molecular confirmation was achieved in 111 patients, while 31 were diagnosed through integrated clinical, radiological, and biochemical criteria. Genetic testing was conducted and analyzed by medical molecular geneticists at the National Research Centre’s molecular diagnostics laboratory, employing the following methods:

1. Targeted Sanger sequencing: for *ARSA* (MLD), *MLC1* (megalencephalic leukoencephalopathy with subcortical cysts, MLC), *ASPA* (Canavan disease), *GALC* (KD), *TREX1* (Aicardi-Goutières syndrome, AGS), *ALDH3A2* (Sjögren-Larsson syndrome, SLS), *ERCC8* (Cockayne syndrome), *PLP1* (Pelizaeus-Merzbacher disease, PMD), *FAM126A* (hypomyelination and congenital cataract, HCC), and *OCLN* (band-like calcification with simplified gyration and polymicrogyria, BLC-PMG).
2. Whole-exome sequencing: for undiagnosed cases (*n* = 60). Genomic DNA of the patients and their available family members was extracted using a standard extraction procedure. A solo exome sequencing was performed using SureSelect Human All Exome 50 Mb Kit (Agilent, Santa Clara, CA, USA) and analyzed on Illumina NovaSeq 6000 (Illumina, San Diego, CA, USA). The obtained sequences were aligned to UCSC **(**University of California, Santa Cruz) human genome GRCh37/hg19, and variants were verified through the GATK pipeline. Identified variants were checked against known public genetic databases like Genome Aggregation Database v.4 (gnomAD, https://gnomad.broadinstitute.org/) dbSNP (http://www.ncbi.nlm.nih.gov/SNP/) and our in-house database. Pathogenicity of the detected variants was predicted using dbNSFP (http://database.liulab.science/dbNSFPconn), which offers scores from 38 different bioinformatic tools, and MutPred2 (http://mutpred.mutdb.org/).

### Classification

A multidisciplinary team of pediatric neurologists and geneticists classified all cases as LDs or GLEs using the standardized GLIA classification system [2].

### Statistical analysis

Descriptive statistics were used to summarize demographic and clinical variables. Categorical data (e.g., sex, consanguinity) are reported as frequencies (percentages), while continuous variables (e.g., age at diagnosis) are presented as mean ± standard deviation (SD). The cumulative childhood incidence of GWMDs was calculated by dividing the total number of confirmed cases (*n* = 142) by the population of children under 18 years in Beni-Suef Governorate during the study period (2019–2023). This value was multiplied by 100,000 to express incidence as cases per 100,000 children. Population denominator data were directly obtained from the Beni-Suef Health Directorate, the local health authority responsible for regional demographic records. Statistical analyses were conducted using IBM SPSS Statistics, Version 26.0 (IBM Corp.).

## Results

### Cohort demographics

During the five-year study period, 142 patients from 113 unrelated families were diagnosed with GWMDs, comprising 56 patients (39.5%) with LDs and 86 patients (60.5%) with GLEs. Rural residence was common in LDs (92.9%) and GLEs (94.2%). Both groups exhibited a male predominance, with LDs at 66.1% and GLEs at 54.7%. Consanguinity rates were 76.8% in LDs and 77.9% in GLEs. A family history of similar GWMDs was observed in 41.1% of LD cases and 55.8% of GLE cases. Demographic, clinical, metabolic assay, and neuroimaging findings are detailed in Tables 1 and 2.

**Table 1:** Demographic, clinical, metabolic assay, and neuroimaging findings in patients with leukodystrophies

Disorder (Total number)	M: F	Age (years)	Age of onset (years)	Age at diagnosis (years)	Neuro-regression	GDD	Seizure	Seizure type	Systemic features	Metabolic assays	Neuroimaging findings	Outcome died: alive
MLC (10)	6:4	1.5–10	0.42–1	1–7	0/10	2/10	5/10	GTC, focal	Nil	Not available (NA)	Diffuse WM T2 hyperintensities with anterior temporal subcortical cysts	0:10
Saposin B-defecient MLD (2)	0:2	5–7	1.5	6	2/2	2/2	0/2	-	Nil	Plasma lyso-Gb3 (12–14.4) ng/ml	Periventricular WM T2 hyperintensities with sparing of subcortical U fibers	0:2
ARSA-related MLD (11)	6:5	2.25–8	1.33–2	0–8	11/11	11/11	2/11	GTC	Nil	Arylsulfatase A Activity: 0–1.8 μmol/gpt/hr	Periventricular WM T2 hyperintensities with sparing of subcortical U fibers	9:2
ALD (6)	6:0	5–9	5–7	5–7	5/6	5/6	1/6	GTC	Adrenal insufficiency, hyperpigmentation	LC/MS:C26: 1.699—2.702, C24/C22 Ratio:1.432- 3.871, C26/C22 Ratio:0.191- 0.219	Bilateral symmetrical parieto-occipital T2 hyperintensities	3:3
Canavan disease (5)	5:0	2.5–7.5	1–2	1.25—2	5/5	5/5	0/5	-	Nil	Urine GCMS: elevated N- acetylaspartic acid	Diffuse WM T2 hyperintensity with involvement of subcortical U fibers, globi pallidi, thalami, and brain stem. Elevated NAA and NAA: creatine ratio by MRS	5:0
Krabbe disease (4)	3:1	0.58–1	0.16–0.5	0.5–1	4/4	4/4	0/4	-	FTT	B-galactocerebrosidase activity = 0.05—0.06 μmol/g.prot/h	Periventricular WM T2 hyperintensities with sparing of subcortical U fibers and with bilateral thalamic calcification on CT brain	4:0
SLS (5)	3:2	3.5—18	0.33- 3	2—16	5/5	5/5	1/5	GTC	Ichthyosis	NA	Periventricular WM T2 hyperintensities	1:4
PMD (2)	2:0	2–3	0.25	0.75	0/2	2/2	0/2	-	Nil	NA	Diffuse T2 hyperintensities of the supra and infratentorial WM	0:2
PMD-like (1)	0:1	5.5	0.083	0.75	0/1	1/1	0/1	-	Nil	NA	Diffuse T2 hyperintensities of the supra and infratentorial WM with involvement of the pons	0:1
HCC (2)	1:1	6–8	0.62	2- 4	0/2	2/2	1/2	GTC	Cataract	NA	Diffuse WM T2 hyperintensities with sparing (or relative sparing) of subcortical WM	0:2
H-ABC (1)	1:0	1.67	1	1.5	0/1	1/1	0/1	-	Nil	NA	Diffuse T2 hyperintensity of the supratentorial WM with atrophy of the cerebellum and basal ganglia	0:1
AGS (6)	3:3	1–7	0–0.33	0.33—5	6/6	6/6	5/6	GTC	FTT, chilblains	NA	Diffuse WM T2 hyperintensities with cerebral atrophy and punctate calcifications in basal ganglia, periventricular WM, and cerebellum on CT brain	5:1
DBP (1)	1:0	0.75	0.021	0.33	0/1	1/1	1/1	Focal	Hepatomegaly	NA	Peritrigonal T2 hyperintensities, hypoplastic corpus callosum, cerebellar atrophy, and polymicrogyria	0:1

Reference Ranges (RR): Arylsulfatase A: Normal > 50 μmol/gpt/hr, Pseudodeficiency 5–50, MLD < 5; Plasma lyso-Gb3 ≤ 1.8 ng/mL; Plasma C26 < 0.57, C24/C22 < 1.2, C26/C22 < 0.03; β-galactocerebrosidase 0.5–4 μmol/g.prot/h

*Abbreviations*: *ALD* adrenoleukodystrophy; *AGS* Aicardi-Goutières syndrome, *ARSA-related MLD* arylsulfatase A-related metachromatic leukodystrophy, *DBP* D-bifunctional protein deficiency, *FTT* failure to thrive; *GTC* generalized tonic–clonic; *H-ABC* hypomyelination with atrophy of the basal ganglia and cerebellum, *HCC* hypomyelination with congenital cataract, *LC/MS* liquid chromatography-mass spectrometry, *GCMC* gas chromatography-mass spectrometry, *MLD* metachromatic leukodystrophy (Saposin B-deficient or ARSA-related), *MLC* megalencephalic leukoencephalopathy with subcortical cysts, *PMD* Pelizaeus-Merzbacher disease, *PMD-like* Pelizaeus-Merzbacher disease-like disorders, *SLS* Sjögren-Larsson syndrome

**Table 2:** Demographic, clinical, metabolic assay, and neuroimaging findings in patients with genetic leukoencephalopathies

Disorder/Total number	M: F	Age (years)	Age of onset (years)	Age at diagnosis (years)	Neuro-regression	GDD	Seizure	Seizure type	Systemic features	Metabolic assays	Neuroimaging findings	Outcome died: alive
Cockayne (18)	8:10	0.5–8.5	0.17–1	0–8.67	6/18	18/18	3/18	GTC	FTT (18), Photosensitivity (17)	NA	Diffuse WM T2 hyperintensities with cerebral and cerebellar atrophy and basal ganglia calcifications on CT brain	3:15
TTD (2)	1:1	3 −7	1	5 −7	0/2	2/2	0/2	-	sparse hair	NA	Diffuse WM T2 hyperintensities	0:2
OCRL (1)	1:0	2.5	0.5	4.5	0/1	1/1	0/1	-	Cataract, tubular dysfunction	NA	Periventricular WM T2 hyperintensities with multiple small cysts and dilated perivascular spaces	0:1
CDCBM14 A (2)	1:1	3–7	1	5–7	0/2	2/2	2/2	GTC, atonic	Nil	NA	Asymmetric patchy foci of cerebral WM T2 hyperintensities with bilateral polymicrogyria	0:2
BLC-PMG (1)	0:1	0.25	0.083	0.42	0/1	1/1	1/1	GTC	Nil	NA	Abnormal gyral pattern, polymicrogyria, thin corpus callosum, WM loss and thalamic, pontine, and subcortical band-like calcification on CT brain	1:0
LAMA2-related CMD (11)	10:1	0.25—7	0.33–7.42	0 −2	0/11	0/11	0/11	-	Nil	NA	Periventricular WM T2 hyperintensities with relative sparing of subcortical white matter	1:10
FKRP related CMD (1)	1:0	1	0	1.67	0/1	0/1	0/1	-	Nil	NA	T2 hyperintensities in the periventricular WM and cerebellar hemispheres, ventriculomegaly, cortical atrophy, and cerebellar cysts	0:1
MC1DN5 (1)	1:0	1.83	1.83	2.83	1/1	1/1	1/1	GTC	Nil	NA	Periventricular WM T2 hyperintensity with cystic lesions in the WM (cavitating leukoencephalopathy)	0:1
PKU (8)	4:4	6–11	0.58–2	1.5–10.08	0/8	8/8	0/8	-	Hypopigmented hair (3)	Plasma phenylalanine 890–1460 μmol/L)	Periventricular WM T2 hyperintensities	0:8
L2HGA (5)	1:4	5.5 −17	3–12	5.67–17.17	4/5	4/5	3/5	GTC, myoclonic	Nil	urine GCMS: elevated 2-hydroxyglutaric acid	Subcortical WM T2 hyperintensities with involvement of basal ganglia and dentate nuclei and sparing of periventricular WM with supratentorial hydrocephalus	0:5
NCL, Infantile onset (5)	2:3	2–4.5	0.75–1.83	2.5–5	5/5	5/5	3/5	GTC, myoclonic	Nil	NA	Periventricular WM T2 hyperintensities with cerebral and cerebellar atrophy	1:4
GM1 gangliosidosis, infantile onset (4)	4:0	0.5–1.17	0.42—1	0.58–1.5	4/4	4/4/	0/4	-	Macular CRS, splenomegaly, excessive Mongolian spots	Beta-galactosidase: < 1—5.4 μmol//h	Cerebral atrophy, diffuse cerebral WM T2 hyperintensity, T2 hypointensity in the thalami, and hyperdense thalami on CT brain	3:1
GM2 gangliosidosis, infantile onset (Tay Sachs) (2)	1:1	1–1.083	0.5–0.83	1.17–1.25	2/2	2/2	1/2	Myoclonic	Macular CRS	Beta-hexosaminidase A:0.15–0.7 μmol/L/h	Cerebral atrophy, diffuse cerebral WM T2 hyperintensity, T2 hypointensity in the thalami, and hyperdense thalami on CT brain	2:0
GM2 gangliosidosis, infantile onset (Sandhoff) (2)	1:1	0.92–1.17	0.5	1.17–1.67	2/2	2/2	0/2	-	Macular CRS, HSM	Beta-hexosaminidase A:0.6–1.5 μmol/L/h total beta-hexosamindase < 0.35—< 0.7 (LOQ) μmol/L/h	Cerebral atrophy, diffuse cerebral WM T2 hyperintensity, T2 hypointensity in the thalami, and hyperdense thalami on CT brain	2:0
Sanfilippo A (4)	3:1	1.42–6.33	1.42–3	1.58–6.5	1/4	4/4	0/4	-	Coarse, HSM, hearing loss	NA	Periventricular WM T2 hyperintensities with supratentorial ventricular dilatation	0:4
Sanfilippo B (3)	1;2	3.5–5	2–2.75	3–4	0/3	3/3	0/3	-	Coarse, HSM	N-acetyl glucosaminidase: 0.08–0.16 μmol/l/h	Periventricular WM T2 hyperintensities with supratentorial ventricular dilatation	0:3
MSD (2)	1:1	2–6.5	2—3	2.5–7	2/2	2/2	0/2	-	Ichthyosis, coarse	NA	Periventricular WM T2 hyperintensities with sparing of subcortical U fibers	0:2
CFD (2)	0:2	1.5—8	1—2	2–8.25	2/2	2/2	2/2	GTC, atonic	Nil	NA	Subcortical WM T2 hyperintensities, cerebral and cerebellar atrophy, and BG calcification on CT brain	1:1
MMA (1)	0:1	3	3	3.17	0/1	1/1	1/1	GTC	Nil	Urine GCMS: increased MMA	Periventricular WM T2 hyperintensities	0:1
MSUD (1)	1:0	0.83	0.17	0.92	1/1	1/1	1/1	GTC	Nil	TMS: valine 519.38 μmol/L leucine/Isoleucine:1340.15 μmol/L	T2 hyperintensity in the deep and periventricular WM, dorsal brain stem, and cerebellum	0:1
Glutaric aciduria type I (2)	1:1	0.58—5	0.33–0.5	0.67–1.25	2/2	2/2	0/2		Nil	TMS: elevated C5DC urine GCMG: increased glutaric acid & 3 hydroxyglutaric acid	Diffuse cerebral atrophy and periventricular WM T2 hyperintensity	0:2
SSADH deficiency (2)	2:0	1—2	0.33–0.5	1.17–2.25	0/2	2/2	1/2	GTC	Nil	Urine GCMC: increased 4-hydroxybutyric acid,	T2 hyperintensities in the globi pallidi and subcortical WM	1:1
Hereditary homocystinuria (2)	1:1	2.5—4	2	3.25–4.75	0/2	2/2	0/2		Nil	Plasma homocysteine > 20—65 μmol/L	Periventricular WM T2 hyperintensities	0:2
MoCD (3)	1:2	0.17–0.83	0	0.17–0.83	0/3	3/3	3/3	GTC, myoclonic	Nil	TMS: Urinary S-Sulphocysteine: 419.8- 1040.6 μmol/mmol creatinine Urinary Xanthine: 424.0 −447.9 μmol/mmol creatinine	T2 extensive hyperintensity of the subcortical and periventricular white matter and basal ganglia, thin corpus callosum, cerebral atrophy, and cystic encephalomalacia	3:0
SOD (1)	0:1	0.17	0	0.17	0/1	1/1	1/1	GTC	Nil	TMS: Urinary S-Sulphocysteine 86.8 μmol/mmol creatinine, Urinary Xanthine 4.9 μmol/mmol creatinine	T2 extensive hyperintensity of the subcortical and periventricular white matter and basal ganglia, thin corpus callosum, cerebral atrophy, and cystic encephalomalacia	1:0

Reference Ranges (RR): Beta-galactosidase (≥ 28.5 µmol/L/h); Beta-hexosaminidase (≥ 2.0 pmol/L/h); N-acetyl glucosaminidase (10–45 µmol/L/h); Plasma phenylalanine (10–150 µmol/L); Plasma valine (20–290 µmol/L), leucine/isoleucine (20–245 µmol/L); Total beta-hexosaminidase (≥ 4.5 pmol/L/h); Urinary S-sulphocysteine (< 10 µmol/mmol creatinine); Urinary xanthine (< 40 µmol/mmol creatinine)

*Abbreviations*: *BLC-PMG* band-like calcification with polymicrogyria and simplified gyral pattern, *CDCBM14 A* complex cortical dysplasia with other brain malformations-14 A, *CFD* cerebral folate deficiency, *CRS* cherry-red spot, *FKRP-related CMD* Fukutin-related protein congenital muscular dystrophy, *FTT* failure to thrive, *GTC* generalized tonic–clonic, *HSM* hepatosplenomegaly, *L2HGA* L-2-hydroxyglutaric aciduria, *LAMA2-related CMD* Laminin alpha-2-related congenital muscular dystrophy, *MC1DN5* mitochondrial complex I deficiency nuclear type 5, *MMA* methylmalonic academia, *MoCD* molybdenum cofactor deficiency, *MSD* multiple sulfatase deficiency, *MSUD* maple syrup urine disease, *NCL* neuronal ceroid lipofuscinosis, *OCRL* oculocerebrorenal syndrome of Lowe, *PKU* phenylketonuria, *SOD* sulfite oxidase deficiency, *SSADH* succinic semialdehyde dehydrogenase deficiency, *TMS* tandem mass spectrometry, *TTD* trichothiodystrophy

### Clinical phenotypes

**Fig. 2:**
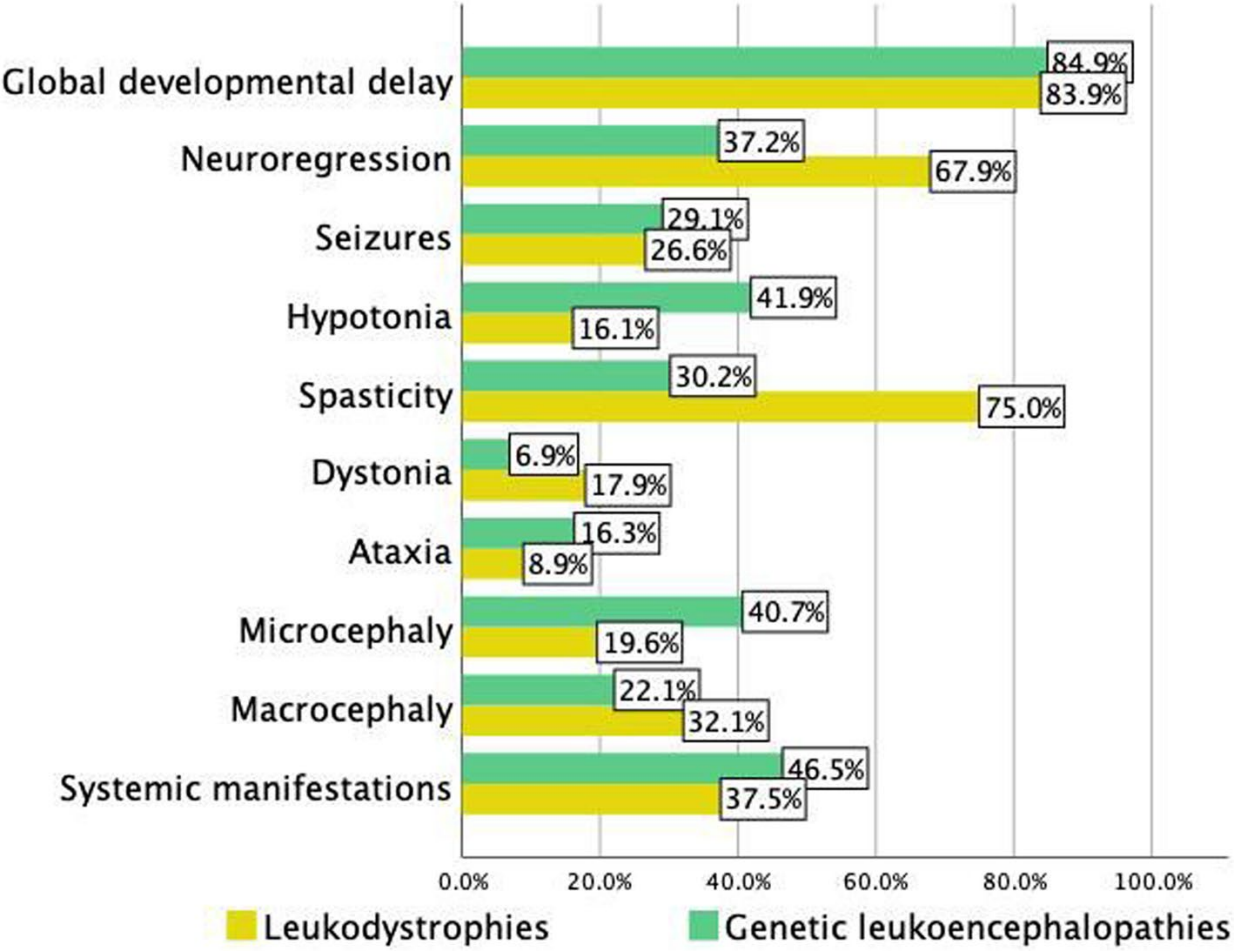
Clinical presentations of patients with genetic white matter disorders in the cohort

Regression of motor or cognitive milestones was significantly more prevalent in LDs (67.9%) than in GLEs (37.2%). In contrast, the incidence of global developmental delay was similar in both groups (LDs: 83.9%; GLEs: 84.9%). Systemic involvement (e.g., hepatosplenomegaly) was observed more frequently in GLE patients (46.5%) compared to LD cases (37.5%). Seizures occurred at comparable rates in both cohorts (LDs: 28.6%; GLEs: 29.1%), with generalized tonic–clonic (GTC) and myoclonic seizures representing the predominant subtypes (Fig. 2).

### Etiologic spectrum

#### Leukodystrophies (LDs)

**Fig. 3:**
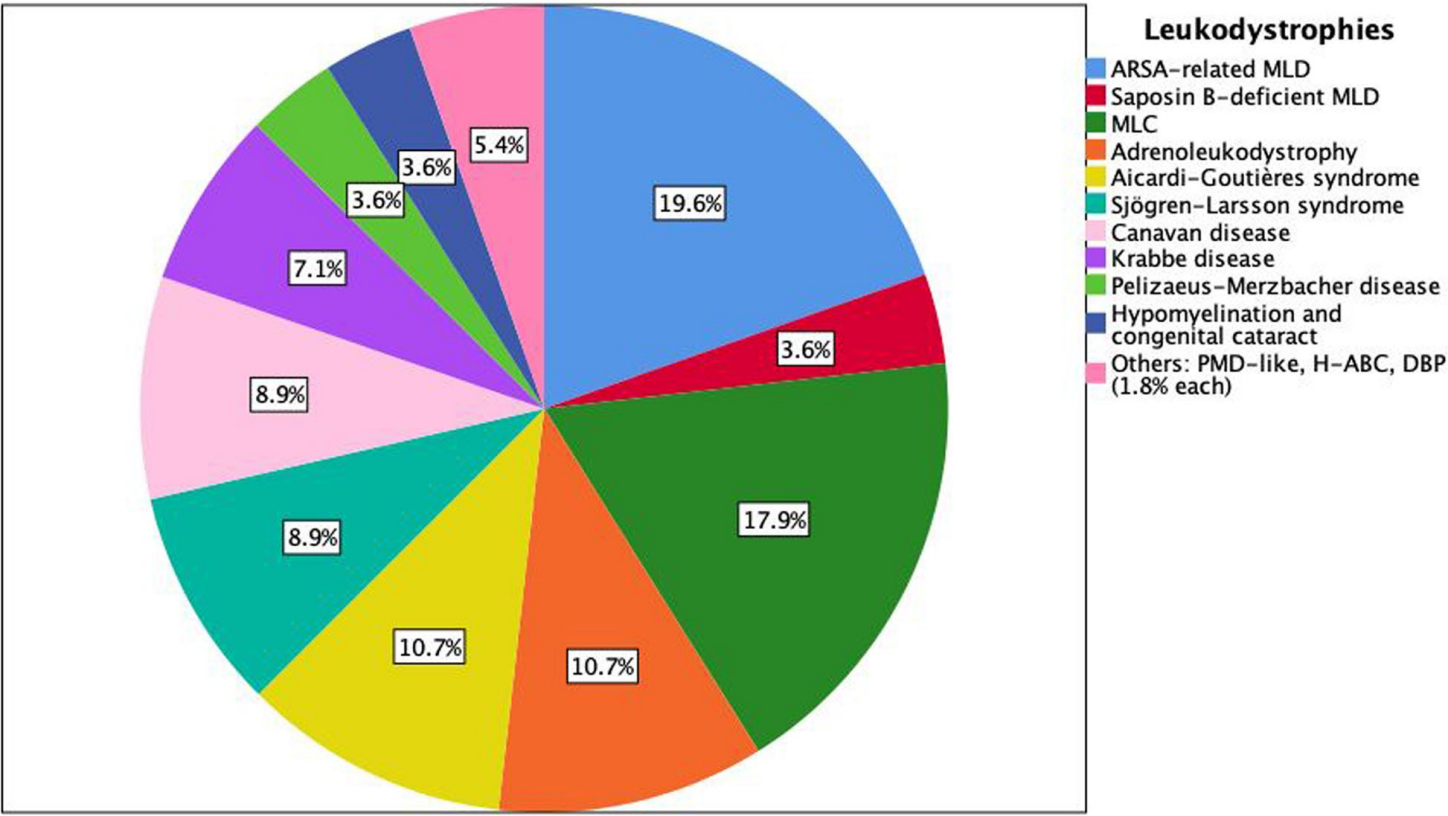
Etiologic spectrum of patients with leukodystrophies. Abbreviations: ARSA-related MLD, arylsulfatase A-related metachromatic leukodystrophy; DBP, D-bifunctional protein deficiency; H-ABC, hypomyelination with atrophy of the basal ganglia and cerebellum; MLC, megalencephalic leukoencephalopathy with subcortical cysts; PMD-like, Pelizaeus-Merzbacher disease-like

MLD was the most prevalent subtype (23.2%, 13/56), followed by MLC (17.9%, 10/56), and AGS and ALD (10.7% each, 6/56**)**. Rare entities, including hypomyelination with atrophy of the basal ganglia and cerebellum (H-ABC) and D-bifunctional protein deficiency, comprised < 5% of cases (Fig. 3).

#### Genetic leukoencephalopathies (GLEs)

**Fig. 4:**
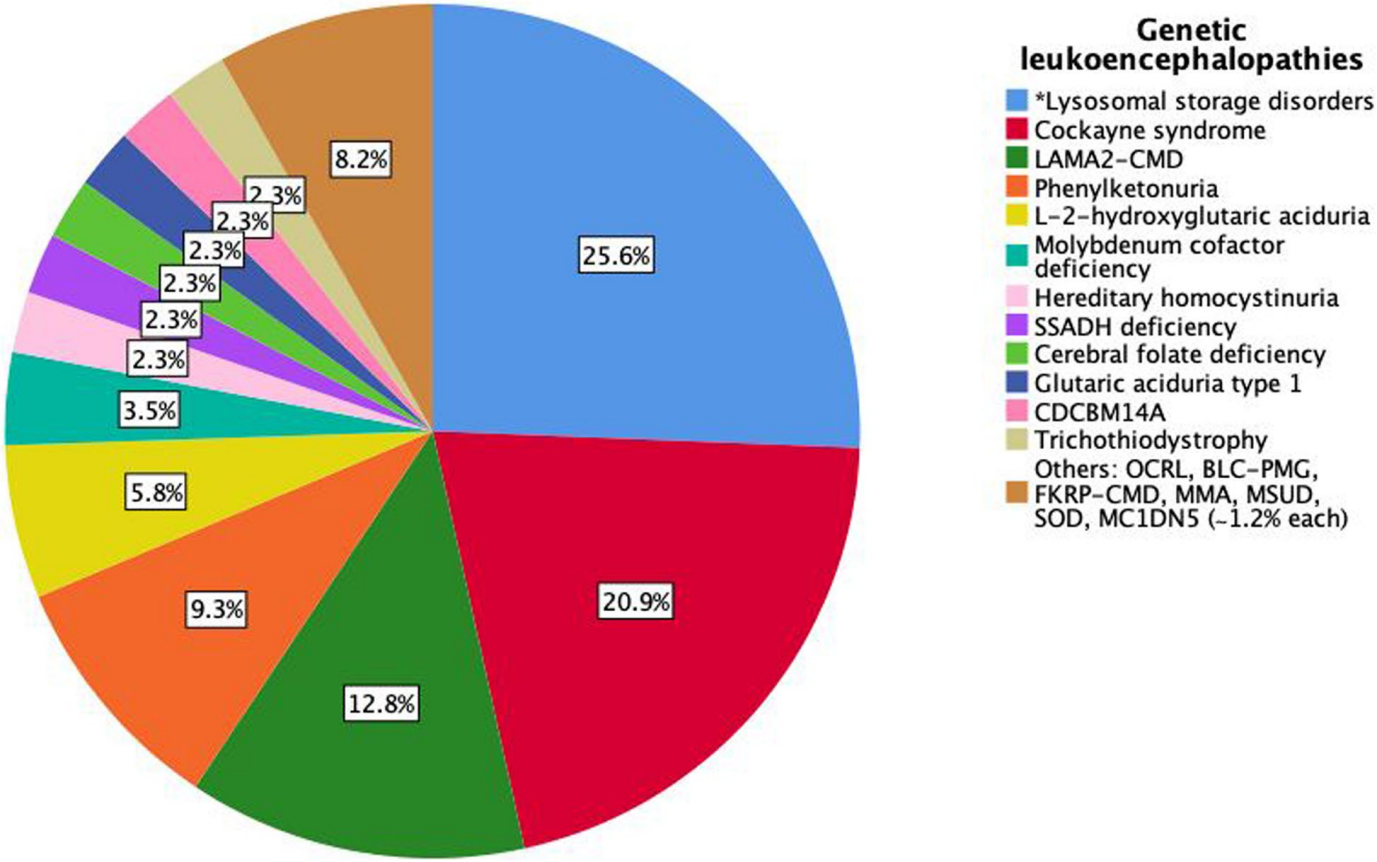
Etiologic spectrum of patients with genetic leukoencephalopathies (GLEs). *Lysosomal storage disorder subtypes (frequency in cohort): Sanfilippo (8.1%), neuronal ceroid lipofuscinoses—infantile onset (5.8%), GM1 gangliosidosis—infantile onset (4.7%), GM2 gangliosidosis—infantile onset (4.7%), multiple sulfatase deficiency (2.3%). Abbreviations: BLC-PMG, band-like calcification with simplified gyration and polymicrogyria; CDCBM14A, complex cortical dysplasia with other brain malformations-14A; FKRP-CMD, Fukutin-related protein congenital muscular dystrophy; LAMA2-CMD, laminin alpha-2-related congenital muscular dystrophy; MC1DN5, mitochondrial complex I deficiency nuclear type 5; MMA, methylmalonic acidemia; MSUD, maple syrup urine disease; OCRL, oculocerebrorenal syndrome of Lowe; SOD, sulfite oxidase deficiency; SSADH deficiency, succinic semialdehyde dehydrogenase deficiency

Lysosomal storage disorders (LSDs) were the most frequent etiology (25.6%, 22/86), with Sanfilippo syndrome (8.1%, 7/86) and infantile-onset neuronal ceroid lipofuscinosis (NCL; 5.8%, 5/86) as predominant subtypes. Cockayne syndrome accounted for 20.9% (18/86) and *LAMA2*-related congenital muscular dystrophy (*LAMA2*-CMD) for 12.8% (11/86), representing significant categories. Rare metabolic disorders, such as molybdenum cofactor deficiency, accounted for < 4% of cases (Fig. 4).

### Genetic findings

Molecular confirmation was achieved in 78.2% of cases (LDs: 44/56; GLEs: 67/86). AR inheritance was predominant, with LDs at 83.9% and GLEs at 98.8%. X-linked recessive disorders were more prevalent in LDs, accounting for 14.3% compared to 1.2% in GLEs. Missense variants constituted the predominant pathogenic alterations in LDs at 56.8%, while nonsense variants were the most prevalent in GLEs at 35.8%. Ten novel variants were identified in LDs (e.g., *ABCD1*, *GALC*) and 21 in GLEs (e.g., *ERCC8*, *LAMA2*), as detailed in Table 3.

**Table 3:** Molecular genetic test results of families within the cohort

Final diagnosis	Gene (N of families)	Genetic variation	Type
Leukodystrophies			
Megalencephalic leukoencephalopathy with subcortical cysts	*MLC1* (4)	-Homozygous c.908_918delinsGCA (p.Val303Glyfs*96)	-Frameshift
		-Homozygous c.908_918delinsGCA (p.Val303Glyfs*96)	-Frameshift
		-Homozygous c.908_918delinsGCA (p.Val303Glyfs*96)	-Frameshift
		- Homozygous c.278C > T (p.Ser93Leu)	-Missense
Saposin B-defecient metachromatic leukodystrophy (MLD)	*PSAP* (1)	- Homozygous c.722G > C (p.Cys241Ser) (2 sibs)	-Missense
Arylsulfatase A-related-MLD	*ARSA* (9)	-Homozygous c.883G > A (p.Gly295Ser)	- Missense
		- Homozygous c.1366C > T (p.Gln456*)	-Nonsense
		-Homozygous c.851A > G (p.Asn284Ser) (3 sibs)	-Missense
		- Homozygous c.851A > G (p.Asn284Ser	-Missense
		- Homozygous c.195C > G (p.Tyr65*)	-Nonsense
		- Homozygous c.712C > T (p.Gln238*)	-Nonsense
		- Homozygous c.195C > G (p.Tyr65*)	-Nonsense
		- Homozygous c.712C > T (p.Gln238*)	-Nonsense
		- Homozygous c.1150G > A (p.Glu384Lys)	-Missense
Adrenoleukodystrophy	*ABCD1*(4)	-Hemizygous **c.1886A > G (p.Asp629Gly) **^**a**^** (2 sibs)**	-Missense
		-Hemizygous c.359G > C (p.Arg120Pro)	-Missense
		-Hemizygous c.430dup (p.Ala144Glyfs*51) ^**a**^** (2 sibs)**	-Frameshift
		- Hemizygous **c.1224 + 1G > A **^**a**^	-Splicing
Canavan disease	*ASPA* (1)	-Homozygous c.502C > T (p.Arg168Cys)	-Missense
Krabbe disease	*GALC* (2)	-Homozygous c.615_619del (p.Tyr205fs*) ^**a**^	-Nonsense
		-Homozygous c.169G > T (p.Gly57Cys)	-Missense
Sjögren-Larsson syndrome	*ALDH3A2* (4)	- Homozygous c.1198G > A (p.Gly400Arg)	-Missense
		- Homozygous c.551C > G (p.Thr184Arg)	-Missense
		- Homozygous c.1094C > T (p.Ser365Leu) (2 sibs)	-Missense
		- Homozygous c.1198G > A (p.Gly400Arg)	-Missense
Pelizaeus-Merzbacher disease (PMD)	PLP1 (2)	-Hemizygous c.169G > T (p.Asp57Tyr)	-Missense
		-Hemizygous **c.527 T > A (p.Ile176Asn) **^**a**^	-Missense
PMD-like	*GJC2* (1)	-Homozygous **c.404G > A (p.Trp135Ter) **^**a**^	-Nonsense
Hypomyelination with congenital cataract	*FAM126A* (1)	-Homozygous c.150_151dup (p.Glu51Valfs*4) ^**a**^** (2 sibs)**	-Frameshift
	*TUBB4A (1)*	- Heterozygous c.721C > T (p.His241Tyr)	-Missense
Aicardi-Goutières syndrome	*TREX1*(4)	- Homozygous c.144dup (p.Thr94Hisfs*53) (2 sibs)	-Frameshift
		- Homozygous c.206_207del (p.Val71Glyf*30) ^**a**^	-Frameshift
		- Homozygous c.144dup (p.Thr94Hisfs*53	-Frameshift
		- Homozygous c.341G > T (p.Arg114Leu)	-Missense
	RNASEH2A (1)	- Homozygous **c.722C > G (p.Ala241Gly **^**a**^	-Missense
D-bifunctional protein deficiency	*HSD17B4* (1)	-Homozygous c.2205del (p.Phe735Leufs*5) ^**a**^	-Frameshift
Genetic Leukoencephalopathies			
Cockayne syndrome	*ERCC8* (12)	-Homozygous c.911G > A(p.Ser304Asn) (2 sibs)	-Missense
		-Homozygous c.966C > A (p.Tyr322*)	-Nonsense
		-Homozygous **deletion of exons 3–12 **^**a**^** (2 sibs)**	-Large deletion
		-Homozygous **c.1016G > A (p.Cys339Tyr) **^**a**^	-Missense
		-Homozygous c.95 T > A (p.Leu32*) ^**a**^	-Nonsense
		-Homozygous c.397C > T (p.Gln133*)	-Nonsense
		-Homozygous c.300C > G (p.Tyr100*) (2 sibs)	-Nonsense
		-Homozygous c.812 T > C (p.Leu271Pro)	-Missense
		-Homozygous c.324 T > A (p.Tyr108*) ^**a**^** (2 sibs)**	-Nonsense
		-Homozygous **c.523 T > C (p.Ser175Pro) **^**a**^	-Missense
		-Homozygous c.300C > G (p.Tyr100*) (2 sibs)	-Nonsense
		-Homozygous c.427_427del (p.Thr143Glnfs*17) ^**a**^** (2 sibs)**	-Frameshift
Trichothiodystrophy	*MPLKIP* (1)	-Homozygous c.221dup (p.Arg77fs) (2 sibs)	-Frameshift
Oculocerebrorenal syndrome of Lowe	*OCRL* (1)	-Hemizygous c.2543 T > A (p.Leu848*) ^**a**^	-Nonsense
CDCBM14A	*ADGRG1* (1)	-Homozygous **c.1141C > T (p.His381Tyr) **^**a**^** (2 sibs)**	-Missense
BLC-PMG	*OCLN* (1)	-Homozygous c.51_730del (p.Lys18_Glu243)	-Large deletion
LAMA2-related congenital muscular dystrophy (CMD)	*LAMA2* (7)	-Homozygous c.443G > A (p.Arg148Gln) (2 sibs)	-Missense
		-Homozygous **c.4718-2A > C **^**a**^	-Splice site
		-Homozygous c.5725G > T (p.Gly1909*) ^**a**^	-Nonsense
		-Homozygous c.6955C > T (p.Arg2319*) (2 sibs)	-Nonsense
		-Homozygous c.6636_6637del (p.Gly2213Ser*)	-Frameshift
		- Homozygous c.2806C > T (p.Gln936*) ^**a**^	-Nonsense
		-Homozygous c.4348C > T (p.Arg1450*) (3 sibs)	-Nonsense
FKRP-related CMD	*FKRP* (1)	-Homozygous c.1364C > A (p.Ala455Asp)	-Missense
MC1DN5	*NDUFS1* (1)	-Double heterozygous **c.283G > A (p.Glu95Lys)**/ **c.462 + 2 T > A **^**a**^	Missense/ Splice site
Phenylketonuria	*PAH* (1)	-Homozygous c.168 + 5G > C	-Splicing
Neuronal ceroid lipofuscinosis, infantile onset	*PPT1* (4)	-Homozygous **c.434-2A > T **^**a**^** (2 sibs)**	-Splicing
		-Homozygous c.871C > T (p.Gln291*)	-Nonsense
		-Homozygous c.799del (p.Asp267Thrfs*5) ^**a**^	-Frameshift
		-Homozygous **c.820dup (p.Met274fs) **^**a**^	-Frameshift
GM1 gangliosidosis, infantile onset	*GLB1* (3)	-Homozygous c.1843del (p.Gln615Argfs*33) ^**a**^	-Frameshift
		-Homozygous c.601C > T (p.Arg201Cys)	-Missense
		-Homozygous c.1883A > G (p.Gln628Arg) (2 sibs)	-Missense
GM2 gangliosidosis, infantile onset (Tay Sachs)	*HEXA* (2)	-Homozygous c.1385A > T (p.Glu462Val)	-Missense
		-Homozygous c.1495C > T (p.Arg499Cys)	-Missense
GM2 gangliosidosis, infantile onset (Sandhoff)	*HEXB* (2)	-Homozygous c.76del (p.Met26Cysfs*5)	-Frameshift
		-Homozygous c.1242G > A (p.Lys414 =)	-Synonymous
Sanfilippo A	*SGSH* (3)	-Homozygous c.948del (p.Asp317Thrfs*96)	-Frameshift
		-Homozygous **c.590 T > C (p.Phe197Ser) **^**a**^** (2 sibs)**	-Missense
		-Homozygous **c.1127 T > G (p.Met376Arg) **^**a**^	-Missense
Sanfilippo B	*NAGLU* (3)	-Homozygous c.2190del (p.Phe731Serfs*76)	-Frameshift
		-Homozygous c.1674C > G (p.Tyr558*)	-Nonsense
		-Homozygous c.889C > T (p.Arg297Ter)	-Nonsense
Multiple sulfatase deficiency	*SUMF1* (1)	-Homozygous **c.859A > G (p.Asn287Asp) **^**a (**^**2 sibs)**	-Missense
Cerebral folate deficiency	*FOLR1* (1)	-Homozygous c.327C > A (p.Cys109*) ^**a**^** (2 sibs)**	-Nonsense
Hereditary homocystinuria	*CBS* (1)	-Homozygous c.1175dup (p.GIn393Alafs*59) ^**a**^** (2 sibs)**	-Frameshift
Molybdenum cofactor deficiency	*MOCS1* (2)	-Homozygous c.253C > T (p.Gln85*) (2 sibs)	-Nonsense
		-Homozygous c.1102 + 1G > A	-Splicing

*Abbreviations*: *BLC-PMG* band-like calcification with simplified gyration and polymicrogyria, *CDCBM14A* complex cortical dysplasia with other brain malformations-14A, *FKRP* Fukutin-related protein, *H-ABC* hypomyelination with atrophy of basal ganglia and cerebellum, *LAMA2* laminin alpha-2, *MC1DN5* mitochondrial complex I deficiency nuclear type 5

^a^Novel variants identified in this cohort (highlighted in bold)

### Outcomes

By the conclusion of the study period, mortality rates reached 39.3% (22/56) among LD patients and 22.1% (19/86) in GLE patients. Progressive neurodegeneration and systemic complications were identified as the primary contributors to mortality in both groups.

## Discussion

This study represents the first cohort assessing the spectrum of LDs and GLEs in Beni-Suef Governorate, Upper Egypt, and, to our knowledge, is the first report from Egypt to describe and classify GWMDs following the GLIA classification [2].

The consanguinity rates in both LDs (76.8%) and GLEs (77.9%) significantly exceed Egypt’s national average (35.3%) [9] and are higher than those reported in rural Upper Egypt (43.6%) [20]. This likely indicates the study cohort’s significant rural predominance (LDs: 92.9%; GLEs: 94.2%), where consanguineous marriages are culturally established and exacerbate the burden of AR disorders. The predominance of AR inheritance (LDs: 83.9%; GLEs: 98.8%) aligns with this trend, as consanguinity increases the homozygosity of recessive alleles. The male predominance in LDs (66.1%) is likely partially due to the inclusion of X-linked disorders (14.3% of LD cases), such as PMD and X-ALD.

The cumulative incidence of GWMDs among children under 18 years in Beni-Suef, Egypt, was estimated at 10.8/100,000 (with LDs accounting for 4.3/100,000), exceeding U.S. estimates (1.2/100,000) [4]. However, it remains markedly lower than Finnish cohorts (30/100,000) 100,000) [5]. This variability likely reflects methodological disparities in case definitions, diagnostic capacity, and patient inclusion, along with genetic and infrastructural factors specific to populations. In Egypt, high levels of consanguinity increase the prevalence of recessive alleles associated with GWMDs. Nevertheless, underdiagnosis caused by delayed referrals, inadequate follow-up, and variability in diagnostic processes obscures the actual epidemiological burden. This paradox, wherein high genetic risk coexists with under-ascertainment, underscores the urgent need for harmonized diagnostic frameworks and a national registry to address diagnostic gaps.

Clinical features aligned with prior reports [21]. However, significant differences were observed between LDs and GLEs. Neuroregression, a hallmark of progressive LDs, was significantly more frequent in LDs than GLEs, consistent with their degenerative nature. In contrast, systemic involvement (e.g., hepatosplenomegaly) and hypotonia predominated in GLEs, reflecting their multisystemic pathology. Systemic manifestations varied across disorders and aided in narrowing the differential diagnosis. These included organomegaly in GM1 and GM2 gangliosidosis, Sanfilippo syndrome and D-bifunctional protein deficiency, failure to thrive (FTT) and photosensitivity in Cockayne syndrome, chilblains in AGS, ichthyosis in multiple sulfatase deficiency** (**MSD) and SLS, and adrenal insufficiency and hyperpigmentation in X-ALD. Ocular manifestations included cataracts found in HCC and oculocerebrorenal syndrome of Lowe (OCRL), as well as cherry red spots (CRS) identified in GM1 and GM2 gangliosidoses.

Using MRI, a pattern recognition approach [22] contributed to the diagnostic workup. Characteristic neuroimaging findings were identified, such as parieto-occipital dysmyelination in ALD, periventricular dysmyelination in MLD, KD, and SLS, subcortical dysmyelination in Canavan disease and L2HGA, and subcortical cysts in MLC. MRS was helpful in the diagnosis of Canavan disease. CT brain imaging demonstrated utility in diagnosing various disorders by identifying specific calcification patterns, such as basal ganglia calcifications in Cockayne syndrome, AGS, and cerebral folate deficiency (CFD); cerebellar calcifications in AGS; and pontine and subcortical band-like calcifications in band-like calcification with simplified gyration and polymicrogyria (BLC-PMG). CT imaging may aid in diagnosing unexplained GWMDs, particularly when associated with microcephaly, prior to molecular testing. Following integrating clinical and radiological data, a provisional diagnosis was established in 93% of LD cases and 65% of GLE patients.

Metabolic testing was crucial in our cohort, providing definitive or suggestive biochemical diagnoses in 50% of LDs and 41.9% of GLEs. Primary diagnoses include aminoacidopathies, organic acidemias, and lysosomal or peroxisomal disorders. Combining clinical features with biochemical and neuroimaging data enhances diagnostic accuracy before molecular testing. However, molecular testing provides definitive diagnoses crucial for prognosis, genetic counseling, and therapeutic planning. A definitive molecular diagnosis was achieved in 78.2% of cases within our cohort (111/142), including 78.6% of LDs (44/56) and 82.7% of GLEs (67/86). Targeted gene sequencing detected 72.7% of LD cases (32 out of 44), in contrast to 27.3% identified through whole-exome sequencing (WES) (12 out of 44). Conversely, WES demonstrated superior performance in GLEs, identifying 71.6% (48 out of 67) compared to 28.4% (19 out of 67) for targeted sequencing. Novel variants comprised 10 in LDs (*ABCD1* [3], *GALC, PLP1, GJC2, FAM126A, TREX1, RNASEH2A, HSD17B4* [1 each]) and 21 in GLEs (*ERCC8* [6], *LAMA2, PPT1* [3 each], *SGSH* [2], and *OCRL, ADGRG1, NDUFS1, GLB1, SUMF1, FOLR1, CBS* [1 each]).

MLD was the commonest LD reported in our work (23.2%), followed by MLC (17.9%), ALD, and AGS (10.7% each). LSDs were the most common GLEs reported in our work (25.6%), followed by Cockayne syndrome (20.9%) and LAMA2-related CMD (12.8%). Our cohort included eight cases of neglected PKU patients exhibiting WM abnormalities, representing 9.3% of GLEs. The increased incidence can be attributed to the prior exclusion of PKU from Egypt’s universal national neonatal screening program until November 2015, leading to untreated patients displaying significant white matter damage—an outcome now preventable due to recent screening reforms.

Two studies conducted in Egypt examined the range of GWMDs. Kamal et al. documented 115 patients with GWMDs from a single referral center in Cairo, identifying Canavan disease as the most common LD (22.6%), followed by hypomyelinating LD-10 (12.2%), MLD (11.3%), and NCL (9.5%), with other less common disorders collectively accounting for the remaining cases [18]. A second study from the same center described 65 LD patients, citing AGS as the most prevalent, followed by MLD, Canavan disease, GM2 gangliosidosis, and vanishing white matter (VWM) disease [17]. The predominance of MLD in our cohort contrasts with the higher prevalence of Canavan disease and AGS observed in previous studies. This may indicate regional genetic diversity or referral bias, given that tertiary centers typically cater to specific populations influenced by diagnostic capabilities or geographic accessibility.

Globally, GWMD distributions exhibit unique genetic and healthcare characteristics. Stellitano et al. from the United Kingdom reported on 803 children with GWMDs, comprising 349 with LDs and 454 with GLEs. MLD represented the most prevalent form of LD, accounting for 22% of cases, followed by X-ALD at 21%, KD at 16%, and various other disorders. Mucopolysaccharidoses (MPS) represented the most prevalent group of GLEs (22% of GLEs), followed by GM1 and GM2 gangliosidoses (20%) and mitochondrial disorders (11%) [6]. In a Finish cohort of GWMDs, ALD was the most common LD (37.5% of LDs). Conversely, mitochondrial disorders were the most common GLEs (23% of GLEs) [5]. In a small Chinese pediatric cohort including 13 patients with GWMDs, X-ALD (30.8%), VWM disease (23.1%), and mitochondrial disorders (15.4%) were the most common [23].

Alfadhel et al. reported on 83 children with LDs in Saudi Arabia, identifying MLD as the most prevalent at 25.3%, followed by VWM disease at 13.3%, peroxisome biogenesis disorder at 12%, AGS at 10.9%, and various other disorders [8]. Mahdieh et al. reported on 152 children with GWMDs from Iran, identifying MLD, Canavan disease, PMD-like, X-ALD, and MLC as the most prevalent LDs. In contrast, Tay-Sachs and Sandhoff diseases were the most common GLEs [24]. This regional variability highlights the significant impact of genetic diversity, healthcare disparities, and public health policies on disease identification. Similarities are present, including the predominance of MLD in our cohort and studies from the UK, Saudi Arabia, and Iran. However, discrepancies, such as the elevated frequency of mitochondrial disorders in Finland, likely arise from founder effects or variations in diagnostic practices. In Egypt, historical gaps in screening have led to delayed PKU diagnosis, affecting cohort composition. This issue is currently being addressed via reforms in neonatal screening. The disparities underscore the necessity for diagnostic protocols and newborn screening programs tailored to regional genetic and infrastructural contexts.

We detected the *ARSA*-related c.851A > G (p. Asn284Ser) variant in four patients from two unrelated families, while the c.712C > T (p. Gln238*) variant was found in two patients from two unrelated families. Amr et al. reported on a cohort of forty-three Egyptian patients with *ARSA*-related MLD, identifying the former and latter variants in eight and six unrelated families, respectively. The presence of these variants in our cohort indicates a founder effect in the Egyptian population [19]. In our four genetically confirmed MLC patients, c.908_918delinsGCA (p. Val303fs) variant was detected in three unrelated families, supporting the founder effect suggested by two earlier studies conducted in Egypt [25, 26].

AGS, representing 11% of our LD cohort, has been reported in Egyptian and other Arab populations. Abdel-Salam et al. emphasized the diagnostic significance of chilblains and further documented unilateral cerebellar hypoplasia and porencephalic cysts in AGS [27, 28]. Al Mutairi et al. reported on 24 AGS patients from four Arab countries [29], and AGS was reported to be the most common LD disorder in Saudi Arabia based on the carrier frequency of the local genomic database in further research [8].

Adrenoleukodystrophy represented 11% of our LD cohort. Only one out of six patients presented early and was referred for SCT. Increased awareness is necessary to facilitate early diagnosis and access to SCT, which may alter disease progression when implemented early [30].

In patients with SLS, we identified the c.1198G > A (p.Gly400Arg) variant in two unrelated families. Abdel-Hamid et al. reported this variant in three unrelated families within a cohort of thirty-five Egyptian patients with SLS [31].

LSDs represented the most common GLEs (25.6%), with Sanfilippo syndrome predominating (8.1%). Almenabawy et al. performed a comprehensive analysis of 34 Egyptian Sanfilippo syndrome patients, identifying 11 novel variants among 25 reported variants [32].

*LAMA2*-CMD accounted for 12.8% of GLEs, highlighting its significance as a key etiological factor. *LAMA2*-CMD induces white matter abnormalities due to laminin-α2 deficiency, frequently presenting diagnostic challenges because of phenotypic overlap with other GWMDs [33], often mimicking other GWMDs and causing a diagnostic challenge. All patients with *LAMA2*-related CMD exhibited hypotonia, normal cognition, and increased creatine phosphokinase (CPK). Another study by Safwat et al. investigated the genetic spectrum of CMDs with brain malformations in Egypt, including five patients (4 families) with *LAMA2*-related CMD. Hypotonia and normal cognition were universally observed in all five patients; however, three demonstrated elevated serum creatine phosphokinase (CPK) levels. The novel variant c.6636_6637del (p.Gly2213Ser*), previously reported in two patients from one family in their study, was also identified in one patient in our cohort [34].

Following the diagnosis of the index patient, proper genetic counseling was offered for all families, including information about inheritance patterns, the methods for future prenatal diagnosis, and assessment of other family members. Current management remains supportive, though recent advances in targeted therapies—including adeno-associated virus (AAV) gene therapy for KD [35] and VWM disease [36], and lentiviral gene therapy for MLD [37, 38] and cerebral ALD [39, 40]—demonstrate therapeutic potential in preclinical and clinical trials. The current study’s findings contribute to understanding GWMDs in Upper Egypt, but it has several limitations. First, the study’s single-center, retrospective design may introduce selection bias, as patients were recruited from a tertiary care hospital in Beni-Suef, which may not fully represent the broader Egyptian population. Second, excluding cases with incomplete data, though methodologically necessary, may have omitted clinically relevant GWMD presentations, potentially narrowing the documented phenotypic spectrum. Finally, limited access to advanced genomic analyses in specific cases may have hindered the identification of novel or complex genetic variants.

## Conclusions

This study represents the first comprehensive characterization of GWMDs in Upper Egypt’s Beni-Suef governorate. A significant finding is the elevated cumulative childhood incidence (10.8 cases per 100,000 children), with 77.4% of cases associated with consanguinity, highlighting the critical necessity for community-based genetic counseling in high-risk populations. Identifying 31 novel variants broadens the mutational spectrum of GWMDs and highlights the significance of population-specific genomic research for improving diagnostics in underrepresented regions. The clinical and radiological features observed align with global reports. However, the unique genetic architecture identified highlights the need for regionally tailored strategies in areas with high consanguinity. These findings support the need for accessible genetic testing, interdisciplinary care pathways, and comprehensive national studies to delineate Egypt’s GWMD landscape. Future initiatives must focus on establishing a national registry for longitudinal tracking, validating the functionality of novel variants, and implementing cost-effective screening protocols in conjunction with public health efforts to improve genetic literacy and reduce diagnostic delays.

## Data Availability

Data and materials are available from the corresponding author upon request.
